# Stem Cells as a Tool for Breast Imaging

**DOI:** 10.1155/2012/814014

**Published:** 2012-07-16

**Authors:** Maria Elena Padín-Iruegas, Rafael López López

**Affiliations:** Hospital Clínico Universitario c/Travesía Choupana s/n. E-15706, Santiago de Compostela, Spain

## Abstract

Stem cells are a scientific field of interest due to their therapeutic potential. There are different groups, depending on the differentiation state. We can find lonely stem cells, but generally they distribute in niches. Stem cells don't survive forever. They are affected for senescence. Cancer stem cells are best defined functionally, as a subpopulation of tumor cells that can enrich for tumorigenic property and can regenerate heterogeneity of the original tumor. Circulating tumor cells are cells that have detached from a primary tumor and circulate in the bloodstream. They may constitute seeds for subsequent growth of additional tumors (metastasis) in different tissues. Advances in molecular imaging have allowed a deeper understanding of the in vivo behavior of stem cells and have proven to be indispensable in preclinical and clinical studies. One of the first imaging modalities for monitoring pluripotent stem cells in vivo, magnetic resonance imaging (MRI) offers high spatial and temporal resolution to obtain detailed morphological and functional information. Advantages of radioscintigraphic techniques include their picomolar sensitivity, good tissue penetration, and translation to clinical applications. Radionuclide imaging is the sole direct labeling technique used thus far in human studies, involving both autologous bone marrow derived and peripheral stem cells.

## 1. Stem Cells

Stem cells are a scientific field of interest mainly due to their therapeutic potential.

The term of stem cells came up to us via histologists in the nineteenth century, who introduced it as a general, abstract term for cells specifically involved in repair or regeneration. With the discovery in the 1950s that bone marrow cells could reconstitute the hematopoietic systems of irradiated individuals, the modern stem cell concept began to crystallize around the experimental procedures of transplantation and reconstitution [1, 2]. The definition for tissue stem cells proposed by Potten and Loeffler was undifferentiated cells (relative to a functional tissue), capable of proliferation and production of a large number of differentiated functional progeny; they have the ability of self-maintenance of their population and for regeneration of the tissue after injury.

This means that stem cells are defined by virtue of their functional attributes and not by an explicit directly observable characteristic. This *functional definition *is relative to the stem cell role linked to the functional tissue regeneration feature. But this definition doesn't give us any characteristic to identify morphologically the stemness [3].

Another point is the fact that it is assumed that stem cells are undifferenctiated and they come from the earlier stages of the development, This means, in a tissue, we can find various types of stem cells, or a stem cell at different points of maturation. (so, gives a possible way to classify descendent transit and mature cells). Most over could be that there are specific differentiation markers which would enable a distinction of stem cells in relation to each other and in relation to the functional cells they are eventually producing.

Flexibility is a key aspect we should include in the definition of stem cells. It may be possible for a stem cell to cease proliferation, that is, become *quiescent*, in which case it does not act as an actual stem cell, but since it can reenter the cycle it has the potential to act as a stem cell. Likewise a transit cell may not normally self-maintain, but may do so under special circumstances, thereby representing a potential stem cell. The recent discovery that stem cell behaviors can be acquired by ordinary cells following the introduction of a small number of genes has intensified its interest.

With this background, all the stem cells are the same type of cell, but as we refer before there are different groups of them depending on the differentiation state; in this sense the three main groups included: toti or pluripotents, multipotents, and commitment cells. The first group are embryonic cells who have the ability to create any kind of tissue. Second group are cells more differentiated, still stem cells, which can create any kind of tissue derived from one of the embrionary layers, endoderm, mesoderm, or ectoderm, for example, mesenchymal stem cells (MSCs). Finally the third group are those who can generate two or more lineages in a tissue, for example, cardiac stem cells (CSCs) [4].

**Figure 1:**
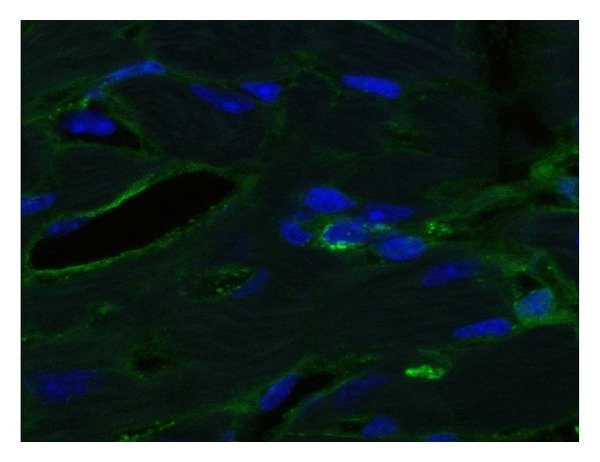
Stem cell niche. C-kit-positive stem cell in the center, surrounded by supporting cells.

Focusing on adult stem cells, as we referred before, they distribute in the different adult tissues, but it's very curious they don't have an aleatory distribution. In fact they use to localize in the most protect areas of the tissue, that is, in heart; they are more abundant in the atria and in the apex, the two localizations were the pressure that supports the tissue is minor or in the ventricular area in nervous system. We can find lonely stem cells, but generally they distribute in niches (Figure 1). We define a niche like type of cells and extracellular substrates that can indefinitely house one or more stem cells (SCs) and control their self-reproduction and production of their progeny *in vivo*. So this means they are specific anatomic locations that regulate how they participate in tissue generation, maintenance, and repair. The niche saves stem cells from depletion, while protecting the host from overexuberant stem cell proliferation. It constitutes a basic unit of tissue physiology, integrating signals that mediate the balanced response of stem cells to the needs of organisms. The simple location of stem cells is not sufficient to define a niche. The niche must have both anatomic and functional dimensions. So functions of niches included: spatial organization, filtration of signals (proliferative, apoptotic …), provided supporting cells, specific unions like cadherins, and determined type division (symmetric or asymmetric) [5].

The niche may also induce pathologies by imposing aberrant function on stem cells or other targets. The interplay between stem cells and their niche creates the dynamic system necessary for sustaining tissues and for the ultimate design of stem cell therapeutics.

 An important aspect of the niche is that determining type division of the stem cells. These cells are very special in this aspect too, because usually when a cell enters in cell cycle producing two daughter cells similar to it. In the case of stem cells this can change, so there are two options as follows.

**Figure 2:**
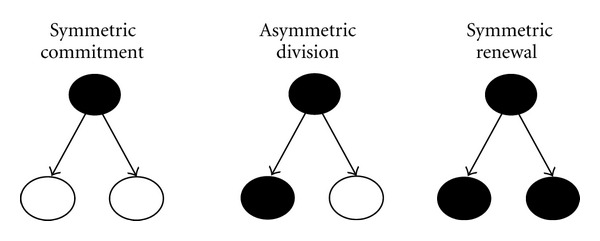
Schema of types division in a stem cell.

- Symmetrical division: the cell gives two daughter cells similar to her, like a regular cell type or two commitment cells.
- Asymmetrical: as the result of a stem cell division we will get two different daughter cells, one similar to the mother and another one that is a commitment cell, so she will be a mature cell (Figure 2).

Once again the regulator of this cellular function is the niche, and this receives the name hypothesis of the free niche. This hypothesis tells us that if there is space for only one stem cell more in the niche, the division will be asymmetrical, but if there is space for two, the division will be symmetrical. Symmetrical division is more frequent in embrionary period and asymmetrical in cellular turnover.

Two proteins are implicated defining the type of division: numb and *α*-adaptin. When the proteins are homogenously distributed in the cell, the division will be symmetrical; instead if protein concentrates in one cellular extreme, division will be asymmetrical [6].

Contrary to what is thought stem cells don't survive forever. They are affected for senescence too, as demonstrated by the Anversa's work [7, 8]. Telomerase shortening, increase in ROS products, and increase in levels of p53, p16 and p66 were demonstrated in stem cells, all of them shared facts with senescent cells.

Many efforts are being made in the study of the mechanism implicated in stem cells, with therapeutic purposes. Fields like degenerative lesions, tisular necrosis, had all their witness in the application of stem cells. In fact, there are several clinical trials focusing on the substitutive therapy with stem cells, in cardiology, neurology, and orthopedic, but still the results are not as good as researchers and clinicians expect.

## 2. Cancer Stem Cells

Recently new discovers can be applied to the stem cells knowledge in the oncology field. Nowadays the theory of the origin of the tumors is in the stem cells is more and more accepted, and every day there are more researchers focusing their efforts implicated in this aspect.

Cancer stem cells (CaSCs) is best defined functionally, as a subpopulation of tumor cells that can enrich for tumorigenic property and can regenerate the heterogeneity of the original tumor in immunocompromised mice. The existence of CaSCs was hypothesized in the 60^'^s, and experimentally isolated in the last decades, first in acute myeloid leukemia and later in solid cancers, such as, breast cancer. Importantly, CaSCs were shown to be resistant to conventional therapies, such as, chemotherapy and radiation. Therefore, the prospective isolation, molecular characterization, and therapeutic targeting of CaSCs in cancer will possibly mark major advances in understanding their pathogenesis.

At the same time, the finding that only a small fraction of the cells within malignant tumors can initiate new tumors upon transplantation has led many cancer biologists to embrace the notion that stem cells are the driving force behind malignancies and to advocate redirecting cancer therapy toward controlling or eradicating stem cells. Clearly, we live in an era of biology when ideas and theories about stem cells are a major part of the intellectual landscape [9].

**Figure 3:**
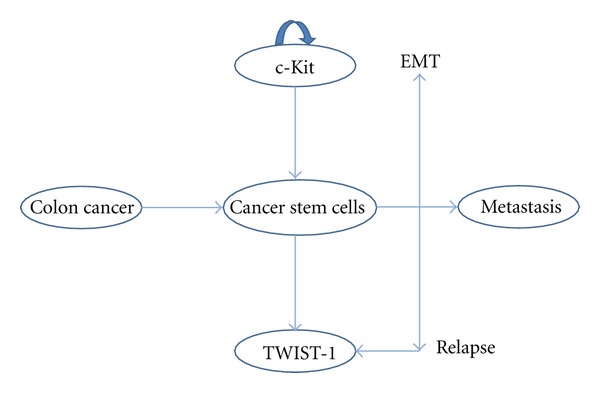
Schema of the theoretical behavior of the stem cells in cancer, its maintenance and transformation to produce distant metastasis.

The hypothesis of cancer is a stem cell disease includes that in a tumour we have, at least, two types of cell population: adult tumour cells and stem cell like, as well as in normal tissue. It has been suggested that cancer is due to an alteration in the normal homeostasis of stem cells. The abundance of cancer stem cells is derived for their symmetric division, and this would be the point to eradicate for cancer treatment. The tumor stem cell hypothesis indicates that this type of cell has all the characteristics of the stem cell: capability of self-renewal, unlimited proliferation potential, multiline differentiation, formation of new adult cells, and asymmetric division, and they are originated of the formation of metastasis, meaning that all the adult tumor cells are coming from this kind of cell (Figure 3). For these properties they are called initiating tumor cells too, and probably, are the responsible for tumors refractoriness and recurrence.

## 3. Circulating Tumor Cells

Another concept is the circulating tumor cells (CTCs) that are cells that have detached from a primary tumor and circulate in the bloodstream. CTCs may constitute seeds for subsequent growth of additional tumors (metastasis) in different tissues [10].

Cells capable of metastasis also acquire the ability to invade another tissue [11]. For epithelial cancers this involves cells undergoing an Epithelial-Mesenchymal Transition (EMT). EMT involves epithelial cells losing their epithelial characteristics and acquiring a more mesenchymal phenotype which occurs as a result of cytoskeletal changes within the cells. These changes allow the cell to acquire a more migratory phenotype [10, 12], increasing the probability of tumour cells entering the blood and lymphatic systems. This process is influenced by chemokines and their receptors which are thought to play an important role in tumour development by influencing tumour transformation, survival, proliferation, invasion, and metastasis and also regulation of angiogenesis and tumour-leukocyte interactions. Despite this, the majority of circulating tumour cells appear to be destroyed [11]. Those that persist may acquire the ability to metastasize and once inside the target organ may undergo Mesenchymal-Epithelial Transition (MET), proliferate and if the environment is conducive the disseminated cells may grow to establish a new tumour thus completing the metastatic process [13].

First evidence indicates that CTCs markers applied in human medicine are conserved in other species. Five of the more common markers including CK19 are also useful to detect CTCs in the blood of dogs with malignant mammary tumors [14].

Standard procedure for isolating circulating stem cells (CTCs) involves cell sorting of a subpopulation on the basis of cell surface markers. Many of these surface markers have been reported as present in the CaSC. These markers change depending on the organ we are considering.

The detection of CTCs may have important prognostic and therapeutic implications but because their numbers can be very small, these cells are not easily detected [15]. Circulating tumor cells are found in different frequencies per mL of whole blood in patients with metastatic disease. To date, a variety of research methods have been developed to isolate and enumerate CTCs [16]. The only USA Food and Drug Administration (FDA) cleared methodology for enumeration of CTCs in whole blood is the CellSearch system. Extensive clinical testing done using this method shows that presence of CTCs is a strong prognostic factor for overall survival in patients with metastatic breast, colorectal, or prostate cancer.

Morphological appearance is judged by human operators and is therefore subjected to large interoperator variation [17]. Several CTCs enumeration methods exist which use morphological appearance to identify CTCs, which may also apply different morphological criteria. A study in prostate cancer showed that many different morphological definitions of circulating tumor cells have similar prognostic value, even though the absolute number of cells found in patients and normal donors varied by more than a decade between different morphological definitions [18].

The behavior of the cells in cancer and metastasis developing will be better known if we will be able to follow these cells.

## 4. Stem Cells Imaging: MRI and Radionuclide Imaging (PET and SPECT)

Over the last decade, advances in molecular imaging have allowed a deeper understanding of the *in vivo* behavior of stem cells and have proven to be indispensable in preclinical and clinical studies.

**Figure 4:**
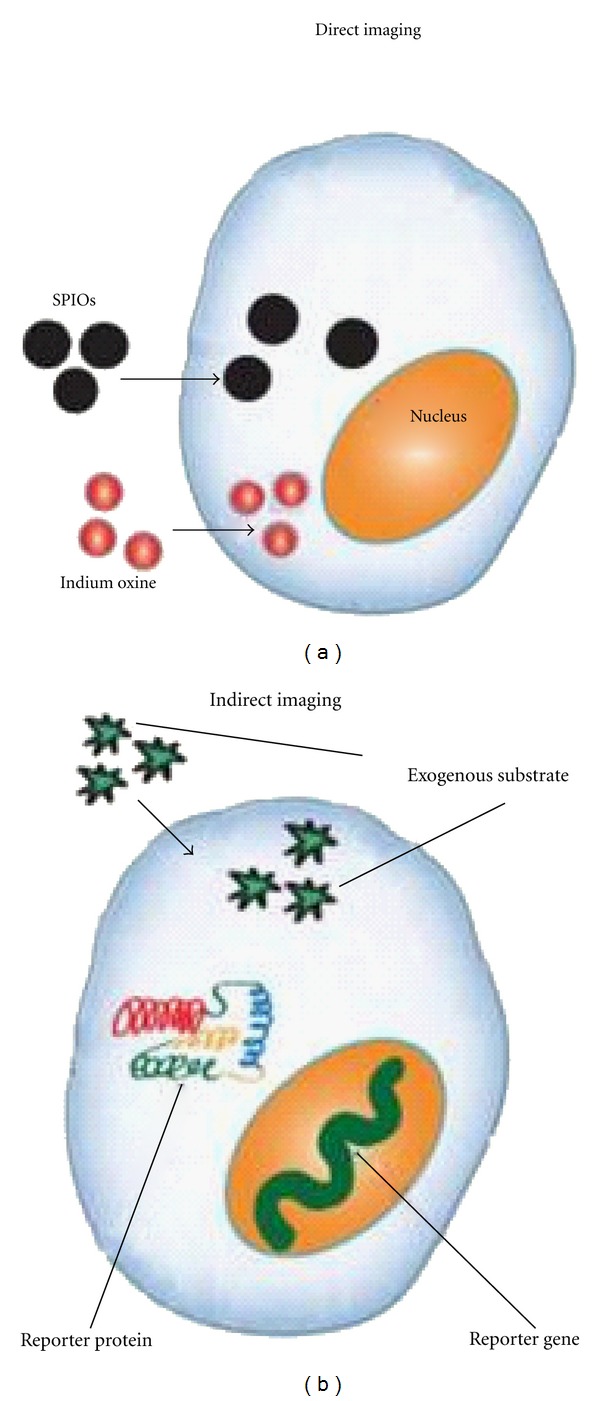
Molecular imaging techniques: direct stem cell labeling and reporter-gene imaging.

There are two main classes of molecular imaging techniques: direct cell labeling and reporter-gene imaging (Figure 4). The former employs contrast agents, such as, magnetic particles, luminescent nanoparticles, or radionuclides to directly label the cell, whereas the latter genetically alters the cell to transcribe and translate a reporter protein. While direct labeling is both straightforward to implement and is commonly used, the contrast signal is diluted with each cellular division and the technique cannot distinguish viable cells from dead cells [19]. Reporter genes, on the other hand, are only expressed by live cells and the signal is propagated by daughter cells [20]. However, reporter gene imaging requires transfection of genetic material using plasmids, retroviral, or viral vectors, which raises the concern of insertional mutagenesis and may necessitate the use of apoptosis-inducing “suicide genes” before possible future use in the clinic [21, 22].

## 5. MRI

As one of the first imaging modalities for monitoring pluripotent stem cells *in vivo*, magnetic resonance imaging (MRI) offers high spatial and temporal resolution to obtain detailed morphological and functional information. It requires the uptake of a contrast agent by the stem cell, the most common of which are superparamagnetic iron oxide (SPIO) nanoparticles. SPIOs can induce changes in T2 relaxivity at nanomolar concentrations [23, 24]. There are two main methods by which stem cells can be directly labeled by SPIOs. One method is magneto operation which involves the coating of anionic SPIOs with cationic transfection agents, such as, protamine sulfate or poly-L-lysine [25]. Stem cells subsequently endocytose the resulting complex during incubation for around 24–48 hours [26]. Although many studies have shown that magneto oporation does not affect cell viability or function at low doses [26–28], there is evidence that high doses can inhibit mesenchymal stem cell (MSC) migration and colony formation ability [29].

Several groups have shown the use of SPIOs for noninvasive MRI of neural stem cell migration, engraftment, and morphological differentiation [30, 31]. The contrast signals in these studies were detected for up to six weeks and the stem cells retained the ability to proliferate and differentiate. Other groups have shown that MRI can be used to track mesenchymal stem cells (MSCs) in cardiac repair after myocardial infarction [32]. Here the signals could be detected long term for three to eight weeks. However, one disadvantage inherent to both SPIO-labeling methods is their inability to distinguish viable cells from dead cells or from scavenging macrophages.

## 6. PET and SPECT

The advantages of radioscintigraphic techniques include their picomolar sensitivity, good tissue penetration, and translation to clinical applications [19]. In fact, radionuclide imaging is the sole direct labeling technique used thus far in human studies, involving both autologous bone marrow-derived stem cells [33] and peripheral hematopoietic stem cells [34–36].

There are two main techniques for radionuclide imaging: positron emission tomography (PET) and single photon emission computed tomography (SPECT). SPECT tracers directly emit a gamma ray in one direction, in contrast to PET tracers, which send two gamma rays in opposite directions and thus possess coincidence detection with a higher spatial resolution. However, SPECT is generally less expensive due to its longer-lived and more readily available radioisotopes.

The most widely used PET isotopes are fluorine-18 (18F), which has a half life of 110 minutes. Copper-64 (^64^Cu) has a much longer half life of 12.7 hours [37]. ^64^Cu can offer a longer duration of *in vivo *visualization of stem cell behavior. ^64^Cu can also be bound to a lipophilic redox-active carrier molecule, pyruvaldehydebis(N4-methylthiosemicarbazone)(PTSM). ^64^Cu-PTSM has been used to image hESCs differentiated towards renal lineages in fetal rhesus monkeys [38] and has been shown to lack adverse cellular effects [39].

The most widely used SPECT radionuclides are indium-111 (^111^In), with a half life of 67 hours and the metastable Technetium-99 m (^99^mTc), with a half-life of 6 hours. While ^111^In provides a longer time window for cell imaging, ^99^mTc can be used in higher doses to improve short-term imaging resolution. Several groups have used ^111^In to image *in vivo *trafficking and biodistribution of MSCs around sites of myocardial injury in the canine [40, 41] and porcine animal models [42]. Human clinical studies have also used ^111^In-oxine [35, 43, 44] to assess stem cell trafficking in acute and chronic myocardial infarction.

Although both PET and SPECT offer great sensitivity, there are several disadvantages to both techniques, including the leakage of radionuclides into nontarget cells [45], limited time window for imaging due to half-life decay, lower spatial resolution as compared to MRI, and the emission of ionizing radiation that may impair stem cell proliferation and survival.

Pluripotent stem cells share many properties in common with cancer cells, including self-renewal, rapid proliferation, lack of contact inhibition, and high telomerase activity [46, 47]. Furthermore, cellular manipulations, such as, the reprogramming of somatic cells into induced pluripotent stem cells (iPSCs), transfection of reporter genes, and overexpression of survival genes, can have unintended tumorigenic side effects. Teratoma formation is another concern, along with its potential to degenerate into malignant teratocarcinomas [46]. Given these risks, understanding stem cell tumorigenicity is of paramount importance for future clinical applications.

One study using a double fusion reporter containing enzyme firefly luciferase (Fluc) and GFP showed that teratoma formation is dependent upon cell number. Assessed over a period of eight weeks, a minimum of 1 × 105 intramyocardially injected hESCs were required to form teratomas in mice [48]. Furthermore, a lower threshold of 1 × 104 cells following hind limb injection was required to form teratomas, providing insight into niche dependency [49]. Since angiogenesis is known to play a major role in tumor growth and development, it is important to investigate not only the tumor itself but also its supporting stroma. One study used sodium iodide symporter (NIS) reporter imaging to show that MSCs actively home in on growing tumors, where they differentiate into vasculature and supporting structures [50]. The upregulation of *α*v*β*3 integrin is also known to play a role in tumor angiogenesis. One study used direct PET imaging with ^64^Cu-DOTA-RGD4 to target *α*v*β*3 integrin, which successfully visualized *in vivo *hESC teratoma formation in the mouse model. These findings show that integrins play a major role in teratoma formation and angiogenesis. Moreover, PET imaging may have promising clinical applicability for monitoring tumorigenicity in humans because BLI lacks the ability to penetrate deep tissues.

Due to the risks of teratoma formation, having a reporter gene that serves as both an imaging modality as well as a failsafe suicide switch would be highly desirable. One study used *HSV1-tk *PET reporter imaging to selectively destroy emerging teratomas with the administration of ganciclovir. Future directions for mitigating the risks of tumorigenicity include not only the use of reporter-suicide genes, but also vector-and transgene-free reprogramming of somatic cells into iPSCs and long-term multimodality imaging capable of observing both emerging tumor cells and their supporting stroma.

Although a great deal of information is already known about the survival, biodistribution, tumorigenicity, and immunogenicity of pluripotent stem cells, significant gaps in knowledge remain. Molecular imaging will continue to play a pivotal role in answering crucial questions about clinical applications as well as in helping us understand the underlying mechanisms of stem cell biology.

In fact, a new imaging agent, radio-labelled hedgehog, detects cancer stem cells, potentially allowing for imaging of “stem cell-like” cancer cells by positron emission tomography (PET) in patients with breast cancer, according to results of a pilot study, presented at the American Association for Cancer Research 101st Annual Meeting 2010. Jennifer Sims-Mourtada, Ph.D., Director of molecular research and development at RadioMedix, Inc., Houston, Texas and colleagues tested the ability to detect breast cancer stem-cell-like populations using a protein, sonic hedgehog that was radiolabeled with the positron emitting isotope gallium-68. Increased activation of the hedgehog pathway is observed in cancer stem cells and aggressive tumours. Binding of the radio-labelled hedgehog to the patched-1 hedgehog receptor on the surface of breast cancer cells occurred, suggesting potential for molecular imaging of breast cancer by PET. A significant increase in binding was observed in cultures enriched for breast cancer stem-like cells.

## 7. Conclusions

More *in vivo *molecular imaging studies must be conducted to confirm long-term survival of these cells. The safety of stem cell therapy in terms of tumorigenicity and immune rejection must also be thoroughly examined. To that end, molecular imaging studies capable of evaluating the risk of cancer formation long-term or assessing methods of immune suppression for viable engraftment are highly valuable.
